# Differential Protein Expression During Growth on Medium Versus Long-Chain Alkanes in the Obligate Marine Hydrocarbon-Degrading Bacterium *Thalassolituus oleivorans* MIL-1

**DOI:** 10.3389/fmicb.2018.03130

**Published:** 2018-12-17

**Authors:** Benjamin H. Gregson, Gergana Metodieva, Metodi V. Metodiev, Peter N. Golyshin, Boyd A. McKew

**Affiliations:** ^1^School of Biological Sciences, University of Essex, Colchester, United Kingdom; ^2^School of Biological Sciences, Bangor University, Bangor, United Kingdom; ^3^School of Natural Sciences, College of Environmental Sciences and Engineering, Bangor University, Bangor, United Kingdom

**Keywords:** *Thalassolituus oleivorans*, alkane degradation, oil pollution, long-chain alkanes, medium-chain alkanes, subterminal oxidation, hydrocarbonoclastic bacteria, shotgun proteomics

## Abstract

The marine obligate hydrocarbonoclastic bacterium *Thalassolituus oleivorans* MIL-1 metabolizes a broad range of aliphatic hydrocarbons almost exclusively as carbon and energy sources. We used LC-MS/MS shotgun proteomics to identify proteins involved in aerobic alkane degradation during growth on medium- (*n*-C_14_) or long-chain (*n*-C_28_) alkanes. During growth on *n*-C_14_, *T. oleivorans* expresses an alkane monooxygenase system involved in terminal oxidation including two alkane 1-monooxygenases, a ferredoxin, a ferredoxin reductase and an aldehyde dehydrogenase. In contrast, during growth on long-chain alkanes (*n*-C_28_), *T. oleivorans* may switch to a subterminal alkane oxidation pathway evidenced by significant upregulation of Baeyer-Villiger monooxygenase and an esterase, proteins catalyzing ketone and ester metabolism, respectively. The metabolite (primary alcohol) generated from terminal oxidation of an alkane was detected during growth on *n-*C_14_ but not on *n-*C_28_ also suggesting alternative metabolic pathways. Expression of both active and passive transport systems involved in uptake of long-chain alkanes was higher when compared to the non-hydrocarbon control, including a TonB-dependent receptor, a FadL homolog and a specialized porin. Also, an inner membrane transport protein involved in the export of an outer membrane protein was expressed. This study has demonstrated the substrate range of *T. oleivorans* is larger than previously reported with growth from *n*-C_10_ up to *n*-C_32_. It has also greatly enhanced our understanding of the fundamental physiology of *T. oleivorans*, a key bacterium that plays a significant role in natural attenuation of marine oil pollution, by identifying key enzymes expressed during the catabolism of *n*-alkanes.

## Introduction

*Thalassolituus oleivorans* MIL-1 is a motile aerobic bacterium belonging to the Gammaproteobacteria class, which was first isolated from seawater and sediment samples collected in Milazzo Harbor, Sicily, Italy, supplemented with the *n*-alkane, tetradecane (*n*-C_14_) (Yakimov et al., 2004). The strain is a typical example of marine obligate hydrocarbonoclastic bacteria (OHCB) which have a highly specialized substrate specificity toward hydrocarbons (Yakimov et al., 2007). Like many other OHCB, such as *Alcanivorax borkumensis* and *Oleispira antarctica*, *T. oleivorans* grows almost exclusively on aliphatic hydrocarbons (Yakimov et al., 1998, 2002). OHCB are present in non-polluted marine environments at low numbers, but following oil spills, they typically bloom and become dominant members of the microbial community (Kasai et al., 2002a,b; Cappello et al., 2007; McKew et al., 2007a; Teramoto et al., 2009; Vila et al., 2010).

The biogeography of *T. oleivorans* shows it is widely distributed all over the world. The RDP (Ribosomal Database Project) and GenBank databases contain 16S rRNA gene sequences of 54 *Thalassolituus-*like bacteria from microbial communities inhabiting both marine (Baltic, Barents, Mediterranean, North, Okhotsk and South China seas, and the Atlantic, Pacific, and Polar Oceans) and terrestrial environments (caves and ground waters) (Yakimov et al., 2007, 2010; Mou et al., 2008). Previous reports have shown that *Thalassolituus*-related species were among the most dominant members of hydrocarbon/petroleum enriched consortia and can outcompete other OHCB such as *Alcanivorax*, which is often the most dominant alkane degrader in marine oil spills (Harayama et al., 2004; Kostka et al., 2011). For example, *Thalassolituus* outcompeted *Alcanivorax* in *n*-C_14_ enriched microcosms even though tetradecane was shown to be the preferred substrate of *Alcanivorax* (Yakimov et al., 2005). *T. oleivorans* became abundant in crude oil-amended North Sea microcosms at both 4°C and 20°C and was in fact the most dominant bacterium at 20°C, with total extractable hydrocarbons 15% their original value, confirming the important role this species plays in crude oil degradation in seawater (Coulon et al., 2007). *T. oleivorans* has also been shown to be the most dominant alkane degrader in both single and mixed alkane seawater microcosms over a range of different length *n*-alkanes from *n*-C_12_ up to *n*-C_32_ (McKew et al., 2007b). Also, in contrast to previous studies, *Thalassolituus*, rather than *Alcanivorax*, was shown to be dominant in crude oil-amended microcosms, suggesting that they are less affected by potentially stressful compounds within crude oil (Hara et al., 2003; Brakstad and Lødeng, 2005; Yakimov et al., 2005). *T. oleivorans* was also one of the dominant species within bacterial communities in deep water samples taken near the oil plume from the Deepwater Horizon oil spill in the Gulf of Mexico (Camilli et al., 2010; Hazen et al., 2010). *Thalassolituus* spp. also dominated the microbial communities present in water samples collected from oil production wells in Canada (Kryachko et al., 2012).

Given the importance of *Thalassolituus* in the attenuation of marine oil pollution, the type strain (*T. oleivorans* MIL-1 DSM 14913^T^) was genome sequenced (Golyshin et al., 2013). *T. oleivorans* has a strong biotechnological potential to be used for bioremediation of marine oil spills due to its high affinity for alkanes, autochthonous marine origin, and critical role in natural cleansing of marine systems. Compared to some physicochemical methods currently used, bioremediation is considered a safer, more efficient and cost-effective alternative for the removal of oil contaminants in the environment (Liu et al., 2011).

Studies investigating the genetics and biochemistry of bacterial alkane degradation have mainly focused on the enzymes involved in the initial step of oxidizing *n-*alkanes, particularly medium-length chains (van Beilen and Funhoff, 2007; Rojo, 2009). Terminal aerobic alkane degradation occurs through sequential oxidation of a terminal carbon initiated by alkane monooxygenases, which produce primary alcohols, and followed by alcohol and aldehyde dehydrogenases, produces the corresponding aldehydes and fatty acids, respectively (Ji et al., 2013). Bacteria capable of degrading medium-chain alkanes frequently contain CYP153-type cytochrome P450s and/or homolog of well characterized integral membrane non-heme iron alkane monooxygenases, such as AlkB (Rojo, 2009; Wang and Shao, 2013). The degradation mechanisms of long-chain alkane oxidation are less well understood. Bacteria degrading long-chain alkanes may contain a long-chain alkane monooxygenase such as AlmA, and/or a thermophilic soluble long-chain alkane monooxygenase, such as LadA from *Geobacillus* (Feng et al., 2007; Throne-Holst et al., 2007). However, the genes that code for these proteins are not present in the *T. oleivorans* genome (Golyshin et al., 2013) and this study provides evidence that suggests an alternative enzyme system used by *T. oleivorans* for long-chain alkane oxidation.

In this study, we used liquid chromatography-tandem mass spectrometry (LC-MS/MS) shotgun proteomics, a powerful tool to simultaneously identify and quantity the differential expression of large sets of proteins. We examined the reorganization of the proteome to identify proteins significantly upregulated whilst growing on either medium- or long-chain alkanes. This has given a unique insight into proteins involved in the transport and degradation of hydrocarbons, for this globally important marine OHCB.

## Results

### Growth of *T. oleivorans* MIL-1

*T. oleivorans* displayed an ability to grow over a wide range of *n*-alkanes of different length and displayed highly similar growth on *n*-alkanes from *n*-C_10_ to *n*-C_32_ (Supplementary Figure 1), with no significant differences in growth rates. After a short lag phase, measurable growth was observed after 3 days on all *n*-alkanes, with cultures displaying a linear growth pattern that entered the stationary phase after 14 days. No growth was observed on the branched alkane pristane. We compared the proteomes during the early stages of growth (day 4) on the medium-chain alkane tetradecane (*n*-C_14_), the long-chain alkane octacosane (*n*-C_28_) compared to the non-hydrocarbon control Tween 80 (one of the very few non-hydrocarbon substrates the strain can grow on).

### Overview of LC-MS/MS Shotgun Proteomic Analysis

12 LC-MS/MS runs were performed consisting of four independent biological replicates of three treatments [medium-chain alkane (*n*-C_14_), long-chain alkane (*n*-C_28_), non-hydrocarbon control (Tween 80)] resulting in 191647 spectral counts that were assigned to 1792 proteins, representing 50% of the total protein-coding genes on the *T. oleivorans* MIL-1 genome (Supplementary Table 1). Almost half (49%) of the spectral counts were assigned to the 100 most abundantly detected proteins and 83% were assigned to the 500 most abundant proteins. The remaining 1292 proteins (representing 17% of spectral counts) were detected with low spectral counts and many not across all biological replicates.

**FIGURE 1:**
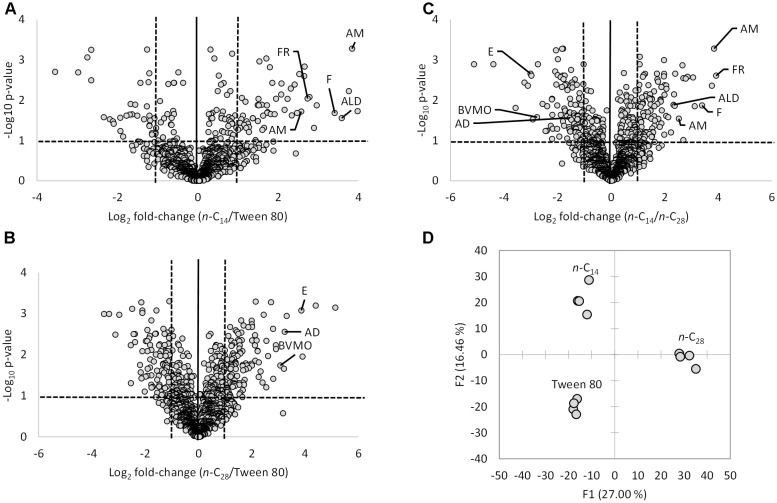
**(A–C)** Volcano plots of normalized LC-MS/MS spectral counts comparing *T. oleivorans* protein biosynthesis during growth on a medium-chain alkane (*n*-C_14_), a long-chain alkane (*n*-C_28_) and a non-hydrocarbon control (Tween 80). Data points above horizontal dashed line represent *P*-values below 0.05. Vertical dashed lines represent a twofold change. Specific proteins involved in the oxidation of alkanes significantly differentially expressed during growth on *n*-C_14_ and *n*-C_28_ are highlighted. AM, alkane monooxygenase; F, ferredoxin; FR, ferredoxin reductase; AD, alcohol dehydrogenase; ALD, aldehyde dehydrogenase; BVMO, Baeyer-Villiger monooxygenase; E, esterase. **(D)** Principal component analysis of replicate *T. oleivorans* proteomes on growth substrates *n*-C_14_, *n*-C_28_, and Tween 80.

A total of 139 proteins were significantly differentially expressed during growth on *n-*C_14_ compared to the non-hydrocarbon control Tween 80 (Figure 1A) with 63% upregulated on *n-*C_14_. 311 proteins were significantly differentially expressed during growth on *n-*C_28_ when compared to Tween 80 (Figure 1B) with 60% upregulated on *n*-C_28_. 234 proteins were significantly differentially expressed during growth on *n*-C_14_ compared to *n*-C_28_ (Figure 1C) with 51% upregulated on *n-*C_14_. Overall the total proteome differed markedly between the three growth substrates with highly similar proteomes between replicates (Figure 1D).

### Terminal Oxidation During Growth on Medium-Chain Alkanes

**FIGURE 2:**
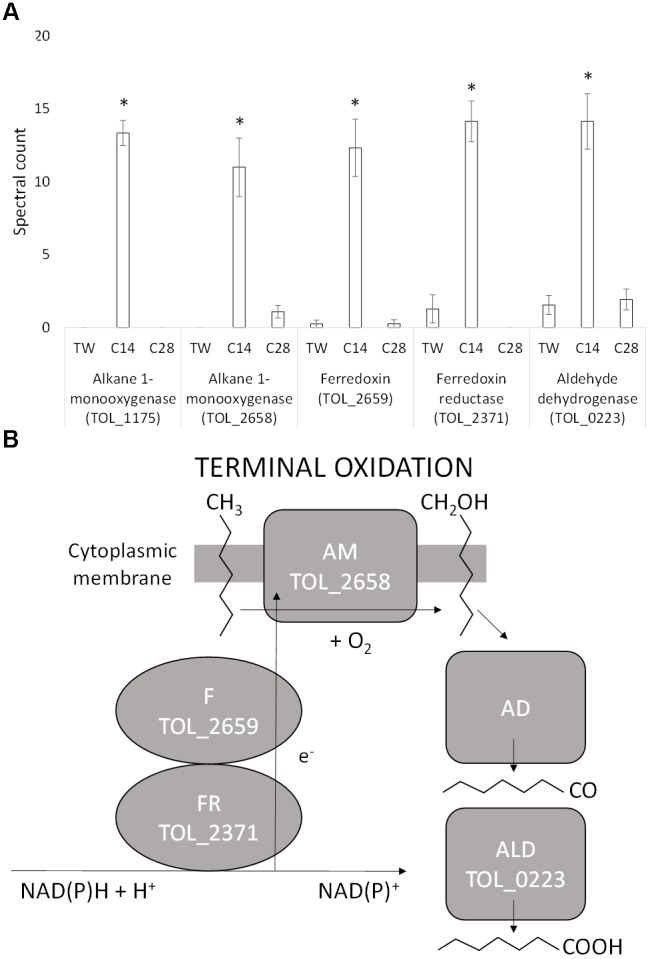
**(A)** Spectral counts (means ± SE; *n* = 4) of differentially expressed terminal alkane oxidation proteins during growth on a medium-chain alkane (*n*-C_14_,), a long-chain alkane (*n-*C_28_,) and a non-hydrocarbon control (Tween 80) in *Thalassolituus oleivorans* MIL-1. ^∗^ denotes spectral counts significantly greater (*P* < 0.05) during growth on *n-*C_14_ relative to growth on *n-*C_28_ and TW. **(B)** Oxidation of an *n-*alkane by the identified proteins in the non-heme iron alkane monooxygenase system. The ferredoxin reductase (FdxR) oxidizes NAD(P)H to NAD(P)^+^. The electrons (e^-^) generated from this are shuttled to the alkane monooxygenase (AM) by the ferredoxin (Fdx). The monooxygenase introduces oxygen into the alkane at the terminal carbon converting it into a primary alcohol. This alcohol is further oxidized to an aldehyde and then to a fatty acid by the alcohol dehydrogenase (AD) and aldehyde dehydrogenase (ALD), respectively.

Five proteins identified as being involved in the terminal oxidation of medium-chain alkanes were significantly differentially expressed during growth on *n*-C_14_ compared to both *n*-C_28_ and Tween 80 (Figure 2A). An alkane 1-monooxygenase (TOL_1175; *P* < 0.001) was exclusively expressed on *n*-C_14_. This protein has a 99% identity to an alkane 1-monooxygenase (R615_11545) from *T. oleivorans* R6-15 and 65% identity to an alkane 1-monooxygenase (OLEAN_C23040) from *Oleispira antarctica* RB-8 encoded by the *alkB2* gene. The expression of a second alkane 1-monooxygenase (TOL_2658; *P* = 0.002) was 11-fold greater in abundance during growth on *n*-C_14_ compared to *n-*C_28_ with no detection in the non-hydrocarbon control, Tween 80. This monooxygenase has 77% identity to an alkane 1-monooxygenase (WP_076515071) from the marine aliphatic alkane-degrader *Oleibacter marinus*. An oxidoreductase (TOL_2659; *P* = 0.001) coded by a gene located adjacent to the alkane 1-monooxygenase was also upregulated in a similar ratio (12-fold higher on *n*-C_14_ compared to both *n*-C_28_ and Tween 80). Domain analysis revealed this oxidoreductase is a ferredoxin that contains a 2Fe–2S iron–sulfur cluster binding domain and it is a member of the Fer2 (PF00111) family. This protein is known to shuttle electrons to the monooxygenase. Expression of a ferredoxin reductase (TOL_2371; *P* = 0.035) was also 14-fold higher during growth on *n*-C_14._ This protein has a 100% and 86% identity to a ferredoxin reductase in *T. oleivorans* R6-15 (W8G014) and *Oleispira marina* (A0A1N7MYW0), respectively. Ferredoxin reductase oxidizes NAD(P)H to NAD(P)^+^ capturing electrons to transfer to the ferredoxin. The Normalized Spectral Abundance Factor (Supplementary Table 1) was higher for the ferredoxin (0.0802%) and ferredoxin reductase (0.1250%) compared to either alkane monooxygenases (TOL_1175-0.0792%; TOL_2658-0.0647%) indicating *T. oleivorans* requires more electron transporters than monooxygenases by themselves. The combination of an alkane monooxygenase and a ferredoxin/ferredoxin reductase indicates a non-heme iron alkane monooxygenase system (Figure 2B) was upregulated during medium-chain alkane degradation. Finally, expression of an aldehyde dehydrogenase (TOL_0223; *P* = 0.001) was at least sevenfold greater during growth on *n-*C_14_ compared to *n-*C_28_ and Tween 80. This combination of proteins makes up a near complete pathway for the terminal oxidation of *n-*alkanes, however, we did not observe the upregulation of a specific alcohol dehydrogenase that would convert the primary alcohol produced by the monooxygenase into the corresponding aldehyde. An alcohol dehydrogenase (TOL_2772) was, however, expressed on all three growth substrates, although it was significantly more abundant during growth on *n*-C_28_ (TOL_2772). In addition to this, an additional three alcohol dehydrogenases (TOL_1420, TOL_2068, TOL_2458) were expressed equally across the three growth substrates and could potentially be constitutively expressed and catalyze the conversion of the primary alcohol to an aldehyde. The metabolite from terminal oxidation of an alkane, the primary alcohol 1-tetradecanol, was detected by GC-MS after 4 days of growth on *n*-C_14_, confirming a terminal oxidation of the alkane, and no secondary alcohols were detected.

### Subterminal Oxidation During Growth on Long-Chain Alkanes

**FIGURE 3:**
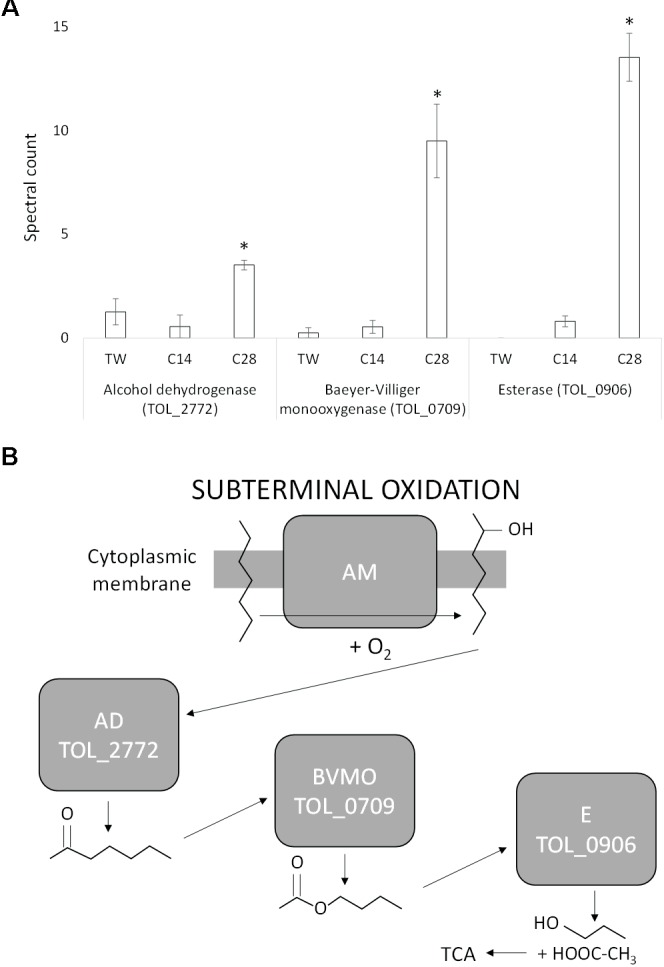
**(A)** Spectral counts (means ± SE; *n* = 4) of differentially expressed subterminal alkane oxidation proteins during growth on a medium-chain alkane (*n*-C_14_,), a long-chain alkane (*n-*C_28_,) and a non-hydrocarbon control (Tween 80) in *Thalassolituus oleivorans* MIL-1. ^∗^ denotes spectral counts significantly greater (*P* < 0.05) during growth on *n-*C_28_ relative to growth on *n-*C_14_ and TW. **(B)** Subterminal oxidation of an *n*-alkane by the identified proteins. An alkane monooxygenase (AM) introduces oxygen into the alkane at a subterminal carbon converting it into a secondary alcohol. The secondary alcohol is converted to the corresponding ketone by an alcohol dehydrogenase (AD). This ketone is then oxidized by a Baeyer-Villiger monooxygenase (BVMO) to render an ester. The ester is then hydrolysed by an esterase (E), generating an alcohol and a fatty acid that then enters the tricarboxylic acid (TCA) cycle.

During growth on *n-*C_28_, a specific subterminal alkane monooxygenase that would oxidize the initial alkane into a secondary alcohol was not identified. However, one of the two alkane monooxygenases (TOL_2658), heavily upregulated during growth via terminal oxidation on *n*-C_14,_ was also expressed during growth on *n*-C_28_ (and not expressed during growth on Tween 80). Three additional proteins identified with a role in the subterminal oxidation of long chain alkanes were, however, specifically significantly upregulated during growth on *n*-C_28_ (Figure 3). The expression of an alcohol dehydrogenase (TOL_2772; *P* = 0.029) was at least 2.5-fold greater during growth on *n-*C_28_ compared to *n*-C_14_ and Tween 80, and this was significantly upregulated together with a flavin-binding family monooxygenase (TOL_0709; *P* = 0.002) that increased 18-fold. TOL_0709 has been identified as a Baeyer-Villiger monooxygenase (BVMO) class enzyme (BVMOs are known to catalyze the conversion of a ketone into an ester via the insertion of an oxygen in a ketone next to the carbonyl atom) due to the presence of two Rossmann fold motifs flanking two BVMO fingerprint sequence motifs (Supplementary Figure 2) (Fraaije et al., 2002; Riebel et al., 2013) and amino-acid sequence homology to known BVMO’s (Supplementary Figures 3, 4). As well as expression of the BVMO, the use of a sub-terminal oxidation pathway is also supported by the expression of an esterase (TOL_0906; *P* < 0.0001) that was 17-fold upregulated during growth on *n*-C_28_ compared to *n*-C_14_ with no expression detected during growth on Tween 80 (Figure 3). An esterase is required to hydrolyse the subsequent ester of a BVMO sub-terminal oxidation to generate an alcohol and a fatty acid. Metabolite analysis also confirmed that no primary (alcohol 1-octacosanol) was detected in any of the 7 days of growth tested.

### Long-Chain Alkane Transport

**FIGURE 4:**
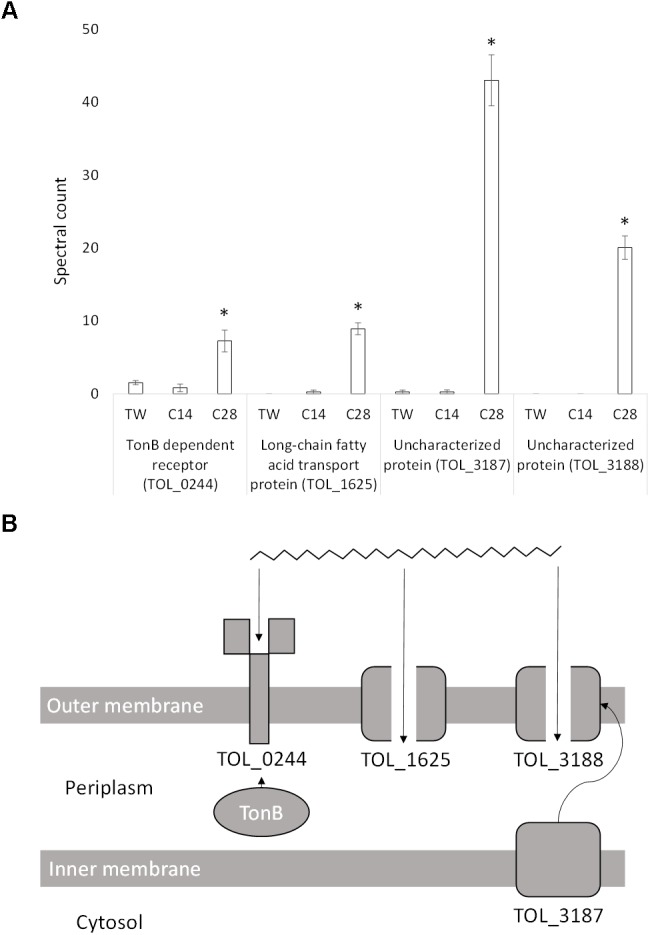
**(A)** Spectral counts (means ± SE; *n* = 4) of differentially expressed long-chain alkane transport proteins during growth on a medium-chain alkane (*n*-C_14_,), a long-chain alkane (*n-*C_28_,) and a non-hydrocarbon control (Tween 80) in *Thalassolituus oleivorans* MIL-1. ^∗^ denotes spectral counts significantly greater (*P* < 0.05) during growth on *n-*C_28_ relative to growth on *n-*C_14_ and TW. **(B)** Proposed uptake of a long chain *n-*alkane by the identified proteins. TonB interacts with TOL_0244 which carries out high-affinity binding and energy dependent uptake of the substrate into the periplasm. In the absence of TonB, the receptor will bind the substrate but not carry out active transport. TOL_1625 is a FadL homolog which allows hydrophobic compounds to cross the cell membrane through diffusion. TOL_3188 is a porin which may facilitate passive uptake of long-chain alkanes and may be transported to the outer membrane by the inner-membrane transport protein TOL_3187.

Due to the size and hydrophobicity of long-chain alkanes (e.g., *n-*C_28_) multiple passive and active transport systems may be required for uptake. Four proteins possibly involved in long-chain alkane transport were significantly differentially expressed during growth on *n*-C_28_ compared to *n*-C_14_ and Tween 80 (Figure 4). Expression of an outer membrane TonB-dependent receptor (TOL_0244; *P* = 0.01) increased sevenfold during growth on *n-*C_28_ compared to Tween 80 (Figure 4A). This receptor has a 40.3% similarity to OmpS (B5T_01485), a TonB-dependent receptor in *Alcanivorax dieselolei*, which was found to be essential for alkane detection and utilization (Wang and Shao, 2014). This suggests the receptor may carry out detection, high-affinity binding and energy-dependent uptake of *n-*C_28_ into the periplasm (Figure 4B).

The expression of a long-chain fatty acid transport protein (TOL_1625; *P* < 0.0001) increased ninefold in the presence of *n-*C_28_ when compared to *n-*C_14_ with no expression detected on Tween 80 (Figure 4A). This protein is a member of the Toluene_X family, which is a family of passive outer membrane transport proteins that include TodX from *Pseudomonas putida* (implicated in toluene catabolism) and FadL, a group of long-chain fatty acids transporters. Given that FadL family proteins have also recently been implicated in alkane transport across the outer membrane (Wang and Shao, 2014), TOL_1625 likely allows passive uptake of *n*-C_28_ into the periplasm (Figure 4B).

The expression of an uncharacterized protein (TOL_3187; *P* < 0.0001) increased 42-fold in the presence of *n-*C_28_ when compared to *n-*C_14_ and Tween 80 (Figure 4A). Domain analysis showed two mycobacterial membrane protein large (MMPL) domains and a LolA-like domain. MMPL proteins are important in substrate transport across the inner membrane to the periplasm. SCOOP analysis in Pfam was used to detect protein families which have a relationship with the MMPL family using sequence information, revealing a relationship with the protein export SecD/SecF family. LolA is required for localization of lipoproteins to the outer membrane. Consensus prediction of GO terms in I-TASSER showed the predicted molecular function of the protein is a transporter (GO:0005215), the biological process is transport (GO:0006810) and the protein is an integral component of a membrane (GO:0016021). Another uncharacterized protein (TOL_3188; *P* < 0.0001) is coded by a gene immediately downstream of TOL_3187 and was expressed exclusively when growing on *n-*C_28_ (Figure 4A). Domain analysis showed that no conserved domains were present. Consensus prediction of GO terms in I-TASSER showed the predicted molecular function is a porin (GO:0015288) and the protein is part of a macromolecular complex (GO:0032991) between two or more proteins. This porin may be transported to the outer membrane via the MMPL protein (TOL_3187) and then facilitate the passage of long-chain alkanes (Figure 4B).

### Chemotaxis Toward Long Chain-Alkanes

**FIGURE 5:**
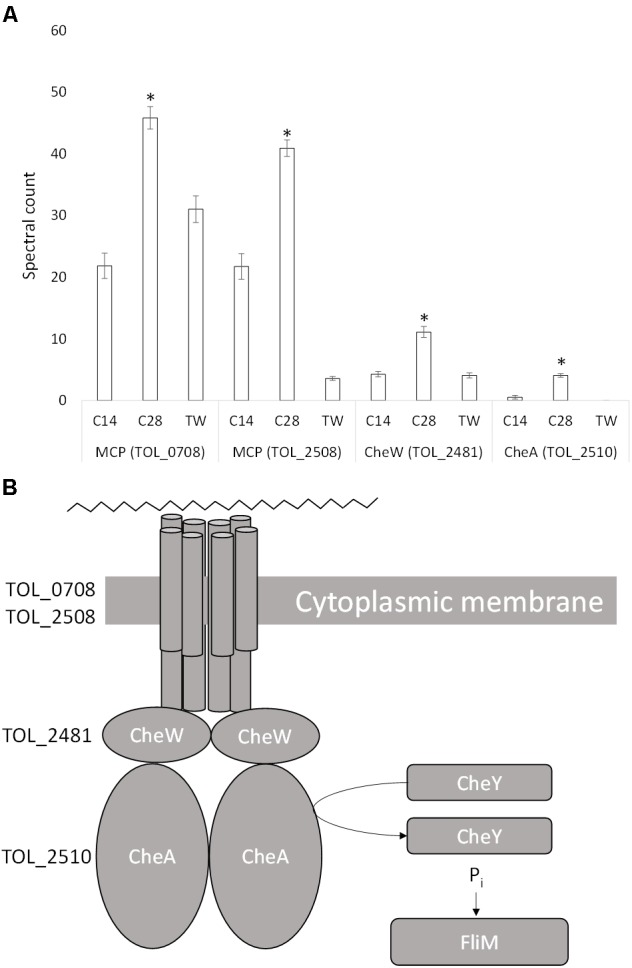
**(A)** Spectral counts (means ± SE; *n* = 4) of differentially expressed chemotaxis proteins during growth on a medium-chain alkane (*n*-C_14_,), a long-chain alkane (*n-*C_28_,) and a non-hydrocarbon control (Tween 80) in *Thalassolituus oleivorans* MIL-1. ^∗^ denotes spectral counts significantly greater (*P* < 0.05) during growth on *n-*C_28_ relative to growth on *n-*C_14_ and TW. MCP, methyl-accepting chemotaxis protein; CheW, purine binding chemotaxis protein; CheA, histidine kinase. **(B)** Proposed chemotactic response of *Thalassolituus oleivorans* MIL-1 to long-chain alkanes. The cytoplasmic side of the MCP dimers interacts with two proteins CheW and CheA. When the MCP is not bound to an attractant, it stimulates CheA to phosphorylate itself using ATP. CheA auto-phosphorylation is inhibited when the attractant is bound to its MCP. CheW physically bridges CheA to the MCPs to allow regulated phosphotransfer to CheY. Phosphorylated CheY phosphorylates the basal body FliM which is connected to the flagellum.

*T. oleivorans* typically has a monopolar, monotrichous flagellum, although a monopolar tuft of four flagella has also been observed (Yakimov et al., 2004). We identified a whole array of corresponding genes spanning the region TOL_2479-TOL_2522 that code for proteins involved in chemotaxis and for components of the flagellum and flagellar motor. Three proteins, coded by genes in this region were significantly upregulated during growth on *n*-C_28_ relative to growth on *n*-C_14_ and Tween 80 (Figure 5). These include a purine-binding chemotaxis protein, CheW (TOL_2481; *P* = 0.001), a methyl-accepting chemotaxis protein, MCP (TOL_2508; *P* < 0.0001) and a histidine kinase, CheA (TOL_2510; *P* < 0.0001). Expression increased 2.5-fold, 2-fold, and 4-fold, respectively, compared to growth on *n*-C_14_ and Tween 80 suggesting a putative role in chemotaxis toward long-chain alkanes. An uncharacterized protein (TOL_0708; *P* = 0.001) was also significantly upregulated, increasing twofold during growth on *n*-C_28_. The gene that codes for this protein is located adjacent to the Baeyer-Villiger monooxygenase (TOL_0709) that was expressed during growth on *n-*C_28_. Domain analysis revealed a HAMP domain and an MCP signal domain, indicating the protein is another MCP. Considering that other M have been detected next to alkane-oxidizing monooxygenases [e.g., *tlpS* in *Pseudomonas aeruginosa* PAO1 (Smits et al., 2003), *alkN* in *Pseudomonas putida* GPo1 (van Beilen et al., 2001)], this suggests that the protein is involved in chemotaxis toward alkanes with a specific role in long-chain alkane metabolism.

## Discussion

*T. oleivorans* is a key member of the marine OHCB characterized by their ability to metabolize hydrocarbons almost exclusively as substrates (Yakimov et al., 2007). OHCB can only use a small number of organic acids or central metabolism intermediates such as acetate, lactate, pyruvate or Tween 40/80 (Yakimov et al., 1998; Golyshin et al., 2003). *T. oleivorans* was capable of growth on every *n*-alkane substrate tested with lengths ranging from *n*-C_10_ to *n*-C_32_. This range of utilizable alkane substrates is far greater than the *n*-C_7_ to *n*-C_20_ range previously documented for *T. oleivorans* (Yakimov et al., 2004; Wentzel et al., 2007) and similar to *Alcanivorax borkumensis* SK2 which is also capable of growth on alkanes up to *n-*C_32_ (Schneiker et al., 2006), although *T. oleivorans* cannot grow on the branched alkane pristane. *T. oleivorans* has a wider substrate range compared to some other OHCB as it is capable of growth on longer-chain alkanes. For example, *Marinobacter hydrocarbonoclasticus* can only metabolize alkanes up to *n-*C_28_ (Klein et al., 2008) and *Oleispira antarctica* can only metabolize alkanes up to *n-*C_24_ (Gregson, unpublished).

### Terminal Oxidation During Growth on Medium-Chain Alkanes

TOL_1175 which was significantly expressed only during growth on *n-*C_14_ codes an alkane monooxygenase which is homologous to AlkB2 from the psychrophilic obligate hydrocarbon degrader *Oleispira antarctica* (OLEAN_C23040, 65% identity). A transcriptional regulator belonging to the GntR family (TOL_1176) was detected in the data set but was not significantly differentially expressed. The gene coding for the regulator is located adjacent to the *alkB2* gene on the genome. This organization of a GntR transcriptional regulator being adjacent to the *alkB2* gene can be seen in other alkane-degrading bacteria, e.g., *P. putida* GPo1, *A. borkumensis* and *A. hongdengensis* (van Beilen et al., 2004; Wang et al., 2010; Sabirova et al., 2011). The electron shuttling proteins rubredoxin and rubredoxin reductase are required for electron transfer to AlkB which it uses for alkane hydroxylation (van Beilen et al., 2006). In general, bacterial cytochrome P450s are the proteins which require ferredoxin and ferredoxin reductase for electron transfer, rather than rubredoxin and rubredoxin reductase. However, TOL_2658 which was exclusively expressed on alkanes and had 11-fold greater expression on *n-*C_14_ compared to *n*-C_28_, encodes an alkane monooxygenase, and TOL_2659, which was expressed in a similar pattern (12-fold higher on *n-*C_14_), encodes a ferredoxin. Ferredoxin and ferredoxin reductase have been shown to functionally replace rubredoxin and rubredoxin reductase *in vitro* (Peterson et al., 1966; Benson et al., 1977). Additionally, analysis of sequenced microbial genomes and metagenomes from terrestrial, freshwater and marine environments found four out of 23 genes encoding multi-domain AlkB system comprised an N-terminal ferredoxin domain, a ferredoxin reductase domain and a C-terminal alkane monooxygenase domain (Nie et al., 2014).

The organization of the genes involved in alkane degradation differs significantly among OHCB (Van Beilen et al., 2003). For example, the genetic organization of *A. borkumensis* is similar to that of *P. putida* Gpo1 where all the proteins required to oxidize an alkane up to the corresponding acyl-CoA derivative are encoded by the *alkBFGHJKL* operon, which includes the alkane monooxygenase and rubredoxins, alcohol and aldehyde dehydrogenases, and an acyl-CoA synthetase (van Beilen et al., 1994, 2001). This pathway appears to have been horizontally transferred across many bacteria (van Beilen et al., 2001). In contrast *T. oleivorans* does not share this level of organization, as many of the proteins involved in alkane degradation that were upregulated are encoded by genes that are distributed throughout the genome rather than on a single operon. In general, alkane-degrading bacteria have multiple alkane monooxygenases which expand the *n*-alkane range of the host strain (van Beilen et al., 2002, 2006). For example, *Alcanivorax borkumensis* contains two *alkB* and three P450 type monooxygenase genes (Schneiker et al., 2006) and multiple monooxygenases exist in the Actinobacteria, *Amycolicicoccus subflavus* DQS3-9A1 and *Rhodococcus* Q15/NRRL B-16531 (Whyte et al., 2002; Nie et al., 2013). When several alkane monooxygenases coexist in a bacterium, they are normally located at different sites in the chromosome, and the alkane-responsive regulators that control expression of the degradation genes (e.g., LuxR/MalR or AraC/XylS) may or may not be adjacent to them (Singh et al., 2012).

### Subterminal Oxidation During Growth on Long-Chain Alkanes

Bacteria degrading long-chain alkanes frequently contain proteins homologous to AlkB a membrane-bound non-heme di-iron monooxygenases found in *Pseudomonas putida* GPo1 which utilizes a terminal oxidation pathway for alkane degradation. For example, *Acinetobacter* sp. M1 contains genes that code for membrane-bound oxygenase complexes involved in long-chain alkane degradation (Tani et al., 2001). This includes *alkMa*, which was induced by long-chain *n-*alkanes greater than *n-*C_22_, and *alkMb*, which was preferentially induced by *n*-alkanes with chain lengths of *n-*C_16_ to *n*-C_22_. *Acinetobacter* sp. DSM 1784 was first reported to have an enzyme involved in the degradation of *n*-alkanes longer than *n*-C_30_, which was named AlmA (Throne-Holst et al., 2007). AlmA homologs have been detected in other OHCB such as *Alcanivorax borkumensis* SK2, *Alcanivorax hongdengensis* A-11-3 and *Alcanivorax dieselolei* B-5 (Rojo, 2009; Wang et al., 2010; Liu et al., 2011). In addition to AlmA, other alkane hydroxylases involved in long-chain *n*-alkane degradation have also been reported, such as a thermophilic soluble long-chain alkane monooxygenase (LadA), which exerts terminal oxidation of alkanes ranging from *n-*C_15_ to *n-*C_36_ in *Geobacillus thermodenitrificans* NG80-2 (Feng et al., 2007; Ji et al., 2013). However, *almA* and *ladA* homologs are not present on the *T. oleivorans’* genome, suggesting an alternative mechanism of long-chain alkane degradation. Several proteins upregulated during growth on *n*-C_28_ (Figure 3) provided strong evidence of a potential subterminal long-chain alkane oxidation pathway. This involves the initial hydroxylation of one of the subterminal carbon atoms in the alkane chain by an alkane monooxygenase (Kotani et al., 2007). Whilst expression of a specific subterminal alkane monooxygenase was not observed during growth on *n*-C_28_, the same monooxygenase that was highly upregulated during terminal oxidation of *n*-C_14_ was also expressed. This suggests the possibility that this enzyme may act in a non-specific regioselective manner and can activate alkanes at either the terminal or sub-terminal position. Such enzymatic non-specificity can result in both primary and secondary alcohols from initial alkane oxidation at both terminal and subterminal carbons (Fredricks, 1967; Markovetz and Kallio, 1971; Singer and Finnerty, 1984).

Secondary alcohols generated can be converted to the corresponding ketone via an alcohol dehydrogenase (TOl_2772 was 2.5-fold upregulated), which would then be subsequently oxidized by the Baeyer-Villiger monooxygenase (BVMO) that was 18-fold upregulated (TOL_0709) to render an ester. The esterase (TOL_0906) would then hydrolyse the ester to generate an alcohol and a fatty acid that enters the tricarboxylic acid (TCA) cycle. Similar esterases are also present in *T. oleivorans* R6-15 (W8G2U9; 100%), *Oleibacter marinus* (WP_076513796.1; 56%) and *Oleispira antarctica* RB-8 (Q6A2S8; 52.5%). Our sequence analysis of the BVMO (TOL_0709) that was upregulated, shows that the amino acid sequence clusters phylogenetically with the BMVO subclass of the group B flavoprotein monooxygenases (Supplementary Figure 3), including the prodrug activator ethionamide monooxygenase (EthA), a *bona fide* BVMO capable of oxidizing several ketones from *Mycobacterium tuberculosis* (Fraaije et al., 2004; Throne-Holst et al., 2007). BLASTp analysis revealed that an identical copy of this BVMO is also present in the other two strains of *Thalassolituus* that recently had their genomes sequenced (*T. oleivorans* K188/CP017810.1; *T. oleivorans* R6-15/CP006829.1). An identical protein sequence (CUS41955.1) has also been detected in a metagenome from a marine hydrothermal vent, and the sequence also shares 71% and 64% identity to putative EthA type BVMOs from the OHCBs *Oleibacter* sp. HI0075 (KZZ04053.1) and *Oleispira antarctica* RB-8 (OLEAN_C10660), respectively, suggesting sub-terminal BMVO type alkane degradation may be more widespread in the marine environment than is currently known. TOL_0709 groups phylogenetically with numerous EthA type BVMO monooxygenases (Supplementary Figure 4) but also has homology with AlmA genes from many species. AlmA has been implicated in both terminal (Wang and Shao, 2014) and subterminal (Minerdi et al., 2012) oxidation of alkanes, but there is still poor understanding of the exact role of AlmA in alkane degradation (Wang and Shao, 2012). All AlmA sequences within the phylogenetic tree (Supplementary Figure 4) do also contain both BMVO fingerprint motifs (Supplementary Figure 2) (Fraaije et al., 2002; Riebel et al., 2013) suggesting that many AlmA proteins may catalyzes subterminal BMVO reactions rather than terminal monooxygenase reactions.

More distantly related BVMOs have been isolated from *Acinetobacter* species such as *Acinetobacter radioresistens* S13 (Minerdi et al., 2012), which was induced during growth in the presence of long-chain alkanes (*n*-C_24_, *n*-C_36_) and shown to subterminally oxidize alkanes. Both *T. oleivorans* MIL-1 and *Acinetobacter radioresistens* S13 are capable of growth on either short- and/or long-chain alkanes as the sole carbon source. They both possess genes coding for terminal alkane hydroxylases homologous to *alkB* and BVMOs homologous to EthA (van Beilen et al., 1992; Minerdi et al., 2012). As seen in *Acinetobacter radioresistens* S13 (Minerdi et al., 2012), *T. oleivorans* MIL-1 appears to differentially express either AlkB or BVMO according to the presence of medium- or long-chain alkanes. Both terminal and subterminal oxidation can coexist in some microorganisms (Rojo, 2009). Subterminal oxidation has been described for both short- and long-chain alkanes as well as for fatty acids (Ashraf et al., 1994; Wentzel et al., 2007). In fatty acids, chain length determines the rate and position of hydroxylation (Van Bogaert et al., 2007). The longer the chain the more the terminal/subterminal oxidation ratio declines. For example, palmitic acid (C_16_H_32_O_2_) and heptadecanoic acid (C_17_H_34_O_2_) are predominantly hydroxylated at the terminal position, whereas no terminal oxidation is observed for oleic acid (C_18_H_34_O_2_). This may also be the case for *n*-alkanes and explain why the subterminal oxidation pathway may potentially is only occur during growth on *n-*C_28_. The production of ketones and esters are impossible in the terminal oxidation pathway so the upregulation of a BVMO and esterase, which catalyze ketone and ester metabolism respectively, and the lack of a primary alcohol metabolite during growth on *n*-C_28_ strongly suggests *T. oleivorans* is using a subterminal oxidation pathway during growth on long-chain alkanes.

### Long-Chain Alkane Transport

Although the genes and proteins that enable the uptake and passage of aromatic hydrocarbons across the bacterial outer membrane have been characterized (for a review, see Wang and Shao, 2013), the transport mechanisms involved in long-chain aliphatic alkane uptake remain unclear. Direct uptake of alkanes from the water phase is only possible for low molecular weight compounds which are sufficiently soluble to facilitate efficient transport into cells (Rojo, 2009). As the molecular weight of the alkane increases its solubility in water decreases (Eastcott et al., 1988). Our data suggests that, to overcome this problem, *T. oleivorans* MIL-1 utilizes a combination of both active and passive transport systems for uptake of alkanes, with higher expression on *n*-C28 compared to the non-hydrocarbon control, including a TonB-dependent receptor (TOL_0244, active, sevenfold increase in *n*-C_28_) a FadL homolog (TOL_1625, passive, ninefold increase in *n*-C_28_) and a specialized porin (TOL_3188, passive, exclusively expressed in *n*-C_28_). An inner membrane transport protein (TOL_3187, active, 42-fold increase in *n*-C_28_) involved in the export of an outer membrane protein was also expressed.

TOL_0244 encodes a TonB-dependent receptor. These receptors are specialized, ligand specific, and use active transport to move compounds against a concentration gradient through the proton-motive force generated through physical interaction with TonB-ExbB-ExbD, an inner membrane complex (Buchanan et al., 1999; Postle and Kadner, 2003). TonB dependent receptor homologs have been detected and implicated in the uptake of alkanes in other OHCB such as OmpS in *Alcanivorax dieselolei* B-5 (Wang and Shao, 2014). The outer membrane protein OmpS (B5T_01485) shares a 40.3% similarity with TOL_0244 and shares conserved domains with a TonB-dependent receptor protein responsible for ferric citrate transport in *Escherichia coli* (EGD69624). OmpS was found to not only detect the presence and utilize alkanes but also trigger the expression of an alkane chemotaxis response. Active transport proteins involved in the uptake of alkanes are not fully understood and only the TonB active transport system had been previously described to be involved.

TOL_1625 encodes a long-chain fatty acid transporter which is a member of the FadL family of proteins. Members of this family are responsible for the passive transport of hydrophobic compounds across the bacterial outer membrane (van den Berg et al., 2004). The first member of the FadL family was isolated from *Escherichia coli* and characterized as a long chain fatty acid transporter (Black et al., 1987). FadL shares some structural similarities with the first known bacterial alkane importer, AlkL, which is part of the *alkBFGHJKL* operon in *Pseudomonas putida* GPo1 (van Beilen et al., 2001; Grant et al., 2014). They both possess a lateral transfer mechanism with high affinity substrate binding causing conformational changes in the N terminus that opens a channel for passive substrate diffusion, allowing the entry of hydrophobic molecules into the outer membrane through the opening in the barrel wall (van den Berg, 2010; Grant et al., 2014). FadL homologs are present in many Gram-negative bacteria which are involved in the biodegradation of xenobiotics (van den Berg et al., 2004). These compounds face the same bacterial cell entry problems as long-chain fatty acids. This suggests hydrophobic compounds enter the cells by a mechanism like that employed for long chain fatty acids by FadL (van den Berg et al., 2004). This theory has been supported by biochemical data on other members of the FadL family including XylN, the xylene transporter from *Pseudomonas putida*, and TbuX, the toluene transporter from *Ralstonia pickettii* PK01 (Kahng et al., 2000; Kasai et al., 2001). FadL homologs have also been detected in other OHCB, such as *A. dieselolei* B-5, which has three outer-membrane proteins belonging to the FadL family shown to be involved in the selective transport of *n*-alkanes (C_8_-C_36_) (Lai et al., 2012; Wang and Shao, 2014).

Proteins that must reach the outer membrane need to be transported from the ribosome where they are synthesized to the inner membrane, where they have to be differentiated from inner-membrane proteins, then transported across the inner membrane into the periplasm, transported through the periplasm and finally assemble into the outer membrane (Reusch, 2012). TOL_3187 is a member of the MMPL family. Members of this family are inner membrane multisubstrate efflux pumps, which belong to the RND (resistance-nodulation-cell-division) permease superfamily of transmembrane transporters (Székely and Cole, 2016). They transport a range of different substrates including trehalose monomycolate, siderophores, phtiocerol dimycocerosate, sulfolipid-1, acylated trehaloses and mycolate ester wax (Jain and Cox, 2005; Seeliger et al., 2012; Varela et al., 2012; Pacheco et al., 2013; Wells et al., 2013; Belardinelli et al., 2014). Substrate transport is driven by the proton motive force (PMF) of the transmembrane electrochemical proton gradient (Chim et al., 2015). MMPL are not restricted to mycobacteria and have been found in related *Streptomyces* and *Rhodococcus* (Deshayes et al., 2010; Letek et al., 2010; Cano-Prieto et al., 2015). SCOOP analysis showed there is a relationship between the MMPL family and the SecDF family. The functional importance of SecDF in protein translocation across the inner membrane was previously shown *in vivo* with SecD and SecF-deficient *Escherichia coli* strains severely defective in protein export (Pogliano and Beckwith, 1994; Nouwen et al., 2005; Hand et al., 2006). This demonstrated bacterial SecD and SecF are required for efficient protein translocation across the inner membrane. The relationship with the SecDF family suggests MMPL may also be involved in export of outer membrane proteins across the inner membrane.

TOL_3187 is also a member of the LolA-like superfamily consisting of two proteins in the Lol system, the periplasmic molecular chaperone LolA and the outer membrane lipoprotein receptor LolB. The Lol system comprises five Lol proteins (A–E) which catalyzes the sorting and outer membrane localization of lipoproteins (Narita et al., 2004; Tokuda and Matsuyama, 2004). Lipoproteins released into the periplasm form a water-soluble complex with the perisplasmic chaperone, LolA (Remans et al., 2010). This LolA-lipoprotein complex crosses the periplasm and then interacts with the outer membrane receptor LolB, which is essential for the anchoring of lipoproteins to the outer membrane (Tsukahara et al., 2009). The Lol system has recently implicated as an alternative to the β-barrel assembly machinery (BAM) for the assembly and insertion of integral outer membrane proteins (Collin et al., 2011; Dunstan et al., 2015; Huysmans et al., 2015; Jeeves and Knowles, 2015). For example, the Pul secretin, a homododecamer of the outer membrane protein PulD, requires the outer membrane lipoprotein PulS for outer membrane localization (Hardie et al., 1996; Nouwen et al., 1999). It has been suggested PulD might “piggy back” on PulS to co-opt LolA for outer membrane localization. The porin (TOL_3188) was exclusively expressed on *n-*C_28_. Most small substrates, with molecular masses below 650 Da, cross the outer membrane via passive diffusion through non-specific porins, such as OmpF or OmpC (Nikaido, 2003). When the size of the substrate is too large for the generalized porins, an alternative specific or specialized porin is used (Davidson et al., 2008). We propose that this specialized porin may be actively transported by TOL_3187 across the inner membrane into the periplasm, then across the periplasm to the outer membrane where it is assembled. Following this it then facilitates passive diffusion of long-chain alkanes.

### Chemotaxis Toward Long Chain Alkanes

Chemotaxis allows the movement of flagellated bacteria toward or away from chemical gradients in the environment and this process plays a role in hydrocarbon degradation by bringing cells into contact with alkanes (Parales and Harwood, 2002). A methyl-accepting chemotaxis protein (MCP) detects the presence of attractant/repellent via a periplasmic sensing domain. Binding of the chemoeffector (alkane) causes the MCP to undergo a conformational change that is transmitted across the inner membrane through to cytoplasmic chemotaxis protein. The signal is transmitted to the flagellar via CheW and the sensor histidine kinase CheA, which can phosphorylate the response regulator CheY. Phosphorylated CheY controls swimming behavior by binding to the flagellar motor to reverse the default direction of flagellar rotation from counter-clockwise to clockwise. This signaling cascade results in movement toward or away from the chemical attractant or repellent. During growth on *n*-C_28_ two MCPs (TOL_0708, TOL_2508), CheW (TOL_2481), and CheA (TOL_2510) were detected suggesting their involvement in chemotaxis toward long-chain alkanes.

Several alkane-specific chemotaxis genes have been found in alkane-degrading bacteria. For example, *tlpS*, which is located downstream of the alkane monooxygenase gene *alkB1* on the *P. aeruginosa* PAO1 genome, has been predicted to encode an MCP that plays a role in alkane chemotaxis (Smits et al., 2003). Similarly *alkN* appears to encode a MCP in *P. putida* GPo1 (van Beilen et al., 2001). Raman microspectroscopy showed chemotaxis in *Acinetobacter baylyi* toward alkanes is highly specific and the chemotaxis proteins therefore follow the same behavior (Li et al., 2017). For example, the coupling protein CheW2 is from *A. dieselolei* was found to be only induced by long-chain alkanes (Wang and Shao, 2014). However, both MCP and CheA were upregulated in response to all types of alkanes, including *n-*C_8_ to *n*-C_32_ and pristane. This data demonstrates an entire chemotaxis complex differentially expressed during growth on long-chain alkanes.

## Conclusion

In conclusion, *T. oleivorans* MIL-1, an important OHCB that can dominate microbial communities following marine oil spills, possess an ability to degrade a wide-range of *n*-alkanes including both medium and long chain lengths. This study has expanded the known substrate range of *T. oleivorans* to include longer *n-*alkanes up to *n*-C32. This study has also significantly enhanced our understanding of the fundamental physiology of *T. oleivorans* MIL-1 by identifying key enzymes involved in both the terminal oxidation of medium-chain alkanes and subterminal oxidation of long-chain alkanes and proteins involved in chemotaxis and transport across the cell membrane of long-chain alkanes. In particular, a potentially novel BVMO system is upregulated for the subterminal oxidation of long-chain alkanes, highlighting an alternative pathway to the currently known AlmA and LadA terminal monooxygenase pathways. Given the homology of the BVMO enzyme TOL_0709 to proteins present in other ubiquitous marine OHCB (e.g., *Oleibacter* and *Oleispira*), this pathway may be widespread in the marine environment.

## Materials and Methods

### Growth Tests

Microcosms were established to determine the alkane substrate range of *T. oleivorans* in sterile 40 ml vials with polytetrafluorethylene (PTFE) lined silicon septa containing 20 ml of ONR7a media (Dyksterhouse et al., 1995). Triplicate single-substrate microcosms were inoculated with *T. oleivorans* MIL-1 (DSM 14913) and enriched separately with the following alkanes at a final concentration of 0.1% w/v: *n*-alkanes: decane (*n-*C_10_), dodecane (*n-*C_12_), hexadecane (*n-*C_16_), eicosane (*n-*C_20_), tetracosane (*n-*C_24_), octacosane (*n-*C_28_), and dotriacontane (*n-*C_32_) (Sigma-Aldrich) or the branched alkane pristane (Sigma-Aldrich). Microcosms were incubated in an orbital shaker (20°C, 60 rpm) for 21 days with the optical density (OD_600_) measured using a NanoDrop 1000 Spectrophotometer. Alkane degradation was measured at day 21 by GC-MS as previously described (McKew et al., 2007b).

### Metabolomics

*T. oleivorans* MIL-1 (DSM 14913) was grown in sterile 160 ml Nunc Cell Culture Treated Flasks containing 100 ml of ONR7a media (Dyksterhouse et al., 1995). Cultures were enriched separately with the medium-chain alkane *n*-tetradecane (*n*-C_14_), or the long-chain alkane *n*-octacosane (*n*-C_28_). Cultures were incubated in an orbital shaker (16°C, 60 rpm) over a period of 7 days. Each day triplicate cultures of *n*-C_14_ and *n*-C_28_ were agitated through water sonication for 30 min and pre-concentrated through solid phase extraction (SPE) by Supel-Select HLB cartridges (Supelco, 200 mg, 6 ml) and eluted with 12 ml of chloroform. Samples were further concentrated through evaporation of the chloroform to 200 μl with nitrogen. The concentrated solvent was analyzed to detect the alcohols produced from the oxidation of the alkanes used as growth substrates. GC-MS analysis was carried out as previously described (McKew et al., 2007b). The mass spectrometer scanned m/z values from 10 to 550, with identification of target analytes based on retention times of analytical standards; 1-tetradecanol, 2-tetradecanol and 1-octacosanol (Sigma-Aldrich).

### LC-MS/MS Shotgun Analysis of *T. oleivorans* MIL-1

#### Culture Conditions

*T. oleivorans* MIL-1 (DSM 14913) was grown in sterile 160 ml Nunc Cell Culture Treated Flasks containing 100 ml of ONR7a media (Dyksterhouse et al., 1995). Cultures were enriched separately with the medium-chain alkane *n*-Tetradecane (*n*-C_14_), or the long-chain alkane *n*-Octacosane (*n*-C_28_), or the non-hydrocarbon control Tween 80 (0.1% w/v) (*n* = 4). Cultures were incubated in an orbital shaker (16°C, 60 rpm) and cells were harvested in early exponential growth phase for protein extraction after 4 days.

#### LC-MS/MS Proteomics

Cells were harvested from 50 ml of each culture by centrifugation (4,600 × *g*, 15 min) and washed in 2 ml of phosphate buffered saline. Total protein was extracted by resuspending the cell pellet in 75 μl of extraction buffer [62.5 mM TRIS, 10% glycerol w/v, 12 mM dithiothreitol (DTT), 2% sodium dodecyl sulfate (SDS) v/v and one Pierce Protease Inhibitor Tablet per 50 ml], heating in a water bath (95°C, 12 min) fully lysing the cells, and then centrifuging (10,500 × *g*, 5 min) to remove cell debris. Protein extracts were visualized by SDS-PAGE and trypsin digestion and LC-MS/MS with a Thermo Fisher hybrid high resolution LTQ Orbitrap instrument was performed as previously described (McKew et al., 2013).

#### MS/MS Analysis

MS/MS analysis was performed in MaxQuant (Cox and Mann, 2008). The LTQ Orbitrap raw data files were first converted to MSM files with the MaxQuant “Quant” module. The open-source search engine Andromeda, which is integrated into MaxQuant, was used to identify peptides in sequence databases by their fragmentation spectra (Cox et al., 2011). Peptides and proteins were filtered at 0.01 false discovery rate (FDR) to obtain the final datasets. Proteins were quantified by counting the number of MS/MS spectra matched to corresponding proteins. Uniprot protein sequences from the *T. oleivorans* MIL-1 genome (Golyshin et al., 2013) were used to perform protein identification. Proteins were validated using the default settings in MaxQuant and Andromeda with a minimum of at least one peptide, but that any such protein had to be unambiguously identified by peptides that were unique to that protein (see full Parameter settings in Supplementary Table 1). Spectral counts were normalized to total spectral counts account for small observed differences between runs (total spectral counts varied between 14707 and 16669). The Normalized Spectral Abundance Factor (number of spectral counts divided by the length of the polypeptide, expressed as percentage for each protein compared to the sum of this ratio for all the detected proteins) was also calculated as longer proteins are expected to produce more peptides. The mass spectrometry proteomics data have been deposited to the ProteomeXchange Consortium (Deutsch et al., 2017) via the PRIDE (Vizcaíno et al., 2016) partner repository with the dataset identifier PXD011824.

#### Statistical and Bioinformatic Analysis

Differential expression analysis was performed by analysis of variance (ANOVA) and Tukey’s HSD test with Benjamini–Hochberg *post hoc* corrections (Benjamini and Hochberg, 1995) within the XLSTAT-Premium Version 2016.1 (Addinsoft) ‘OMICs’ package. All proteins significantly (*P* < 0.05) upregulated during growth on a substrate compared to another were subjected to a BLAST (Basic Local Alignment Search Tool) (Altschul et al., 1990) against the NCBI nr database. Protein family and domain analysis was carried out in Pfam v30.0 (Finn et al., 2016). SCOOP (Simple Comparison of Outputs Program) (Bateman and Finn, 2007) was used to detect relationships between families in the Pfam database. Proteins were assigned to functional families by hierarchical classification of protein domains based on their folding patterns in CATH v4.1 (Class, Architecture, Topology, Homology) (Sillitoe et al., 2015). Full length secondary and tertiary structure predictions, functional annotations on ligand-binding sites, enzyme commissions numbers and gene ontology terms were generated using the I-TASSER SERVER (Zhang, 2008).

## Author Contributions

BG and BM designed the experiments. BG, GM, and MM conducted the experiments and acquired the data. BG, BM, and PG analyzed the data. BG wrote the manuscript. BM, GM, MM, and PG edited the manuscript. BM and PG acquired the funding.

## Conflict of Interest Statement

The authors declare that the research was conducted in the absence of any commercial or financial relationships that could be construed as a potential conflict of interest.

## References

[B1] Altschul S. F. Gish W. Miller W. Myers E. W. Lipman D. J. (1990). Basic local alignment search tool. *J. Mol. Biol.* 215 403–410. 10.1016/S0022-2836(05)80360-2 2231712

[B2] Ashraf W. Mihdhir A. Murrell J. C. (1994). Bacterial oxidation of propane. *FEMS Microbiol. Lett.* 122 1–6. 10.1111/j.1574-6968.1994.tb07134.x 7958761

[B3] Bateman A. Finn R. D. (2007). SCOOP: a simple method for identification of novel protein superfamily relationships. *Bioinformatics* 23 809–814. 10.1093/bioinformatics/btm034 17277330 PMC2603044

[B4] Belardinelli J. M. Larrouy-Maumus G. Jones V. Sorio de Carvalho L. P. McNeil M. R. Jackson M. (2014). Biosynthesis and translocation of unsulfated acyltrehaloses in *Mycobacterium tuberculosis*. *J. Biol. Chem.* 289 27952–27965. 10.1074/jbc.M114.581199 25124040 PMC4183827

[B5] Benjamini Y. Hochberg Y. (1995). Controlling the false discovery rate: a practical and powerful approach to multiple testing. *J. R. Stat. Soc. Ser. B* 57 289–300. 10.2307/2346101

[B6] Benson S. Fennewald M. Shapiro J. Huettner C. (1977). Fractionation of inducible alkane hydroxylase activity in *Pseudomonas putida* and characterization of hydroxylase-negative plasmid mutations. *J. Bacteriol.* 132 614–621. 410794 10.1128/jb.132.2.614-621.1977 PMC221903

[B7] Black P. N. Said B. Ghosn C. R. Beach J. V. Nunn W. D. (1987). Purification and characterization of an outer membrane-bound protein involved in long-chain fatty acid transport in *Escherichia coli*. *J. Biol. Chem.* 262 1412–1419. 3027089

[B8] Brakstad O. G. Lødeng A. G. G. (2005). Microbial diversity during biodegradation of crude oil in seawater from the North Sea. *Microb. Ecol.* 49 94–103. 10.1007/s00248-003-0225-6 15883864

[B9] Buchanan S. K. Smith B. S. Venkatramani L. Xia D. Esser L. Palnitkar M. (1999). Crystal structure of the outer membrane active transporter FepA from *Escherichia coli*. *Nat. Struct. Biol.* 6 56–63. 10.1038/4931 9886293

[B10] Camilli R. Reddy C. M. Yoerger D. R. Van Mooy B. A. S. Jakuba M. V. Kinsey J. C. (2010). Tracking hydrocarbon plume transport and biodegradation at Deepwater Horizon. *Science* 330 201–204. 10.1126/science.1195223 20724584

[B11] Cano-Prieto C. García-Salcedo R. Sánchez-Hidalgo M. Braña A. F. Fiedler H.-P. Méndez C. (2015). Genome mining of *Streptomyces* sp. Tü 6176: characterization of the nataxazole biosynthesis pathway. *ChemBioChem* 16 1461–1473. 10.1002/cbic.201500153 25892546

[B12] Cappello S. Denaro R. Genovese M. Giuliano L. Yakimov M. M. (2007). Predominant growth of *Alcanivorax* during experiments on “oil spill bioremediation” in mesocosms. *Microbiol. Res.* 162 185–190. 10.1016/j.micres.2006.05.010 16831537

[B13] Chim N. Torres R. Liu Y. Capri J. Batot G. Whitelegge J. P. (2015). The structure and interactions of periplasmic domains of crucial MmpL membrane proteins from *Mycobacterium tuberculosis*. *Chem. Biol.* 22 1098–1107. 10.1016/j.chembiol.2015.07.013 26278184 PMC4546533

[B14] Collin S. Guilvout I. Nickerson N. N. Pugsley A. P. (2011). Sorting of an integral outer membrane protein via the lipoprotein-specific Lol pathway and a dedicated lipoprotein pilotin. *Mol. Microbiol.* 80 655–665. 10.1111/j.1365-2958.2011.07596.x 21338419

[B15] Coulon F. McKew B. A. Osborn A. M. McGenity T. J. Timmis K. N. (2007). Effects of temperature and biostimulation on oil-degrading microbial communities in temperate estuarine waters. *Environ. Microbiol.* 9 177–186. 10.1111/j.1462-2920.2006.01126.x 17227422

[B16] Cox J. Mann M. (2008). MaxQuant enables high peptide identification rates, individualized p.p*.b.-*range mass accuracies and proteome-wide protein quantification. *Nat. Biotechnol.* 26 1367–1372. 10.1038/nbt.1511 19029910

[B17] Cox J. Neuhauser N. Michalski A. Scheltema R. A. Olsen J. V. Mann M. (2011). Andromeda: a peptide search engine integrated into the MaxQuant environment. *J. Proteome Res.* 10 1794–1805. 10.1021/pr101065j 21254760

[B18] Davidson A. L. Dassa E. Orelle C. Chen J. (2008). Structure, function, and evolution of bacterial ATP-binding cassette systems. *Microbiol. Mol. Biol. Rev.* 72 317–364. 10.1128/MMBR.00031-07 18535149 PMC2415747

[B19] Deshayes C. Bach H. Euphrasie D. Attarian R. Coureuil M. Sougakoff W. (2010). MmpS4 promotes glycopeptidolipids biosynthesis and export in *Mycobacterium smegmatis*. *Mol. Microbiol.* 78 989–1003. 10.1111/j.1365-2958.2010.07385.x 21062372

[B20] Deutsch E. W. Csordas A. Sun Z. Jarnuczak A. Perez-Riverol Y. Ternent T. (2017). The ProteomeXchange consortium in 2017: supporting the cultural change in proteomics public data deposition. *Nucleic Acids Res.* 45 D1100–D1106. 10.1093/nar/gkw936 27924013 PMC5210636

[B21] Dunstan R. A. Hay I. D. Wilksch J. J. Schittenhelm R. B. Purcell A. W. Clark J. (2015). Assembly of the secretion pores GspD, Wza and CsgG into bacterial outer membranes does not require the Omp85 proteins BamA or TamA. *Mol. Microbiol.* 97 616–629. 10.1111/mmi.13055 25976323

[B22] Dyksterhouse S. E. Gray J. P. Herwig R. P. Lara J. C. Staley J. T. (1995). *Cycloclasticus pugetii* gen. nov., sp. nov., an aromatic hydrocarbon-degrading bacterium from marine sediments. *Int. J. Syst. Bacteriol.* 45 116–123. 10.1099/00207713-45-1-116 7857792

[B23] Eastcott L. Shiu W. Y. Mackay D. (1988). Environmentally relevant physical-chemical properties of hydrocarbons: a review of data and development of simple correlations. *Oil Chem. Pollut.* 4 191–216. 10.1016/S0269-8579(88)80020-0

[B24] Feng L. Wang W. Cheng J. Ren Y. Zhao G. Gao C. (2007). Genome and proteome of long-chain alkane degrading *Geobacillus thermodenitrificans* NG80-2 isolated from a deep-subsurface oil reservoir. *Proc. Natl. Acad. Sci. U.S.A.* 104 5602–5607. 10.1073/pnas.0609650104 17372208 PMC1838512

[B25] Finn R. D. Coggill P. Eberhardt R. Y. Eddy S. R. Mistry J. Mitchell A. L. (2016). The Pfam protein families database: towards a more sustainable future. *Nucleic Acids Res.* 44 D279–D285. 10.1093/nar/gkv1344 26673716 PMC4702930

[B26] Fraaije M. W. Kamerbeek N. M. Heidekamp A. J. Fortin R. Janssen D. B. (2004). The prodrug activator EtaA from *Mycobacterium tuberculosis* is a Baeyer-Villiger monooxygenase. *J. Biol. Chem.* 279 3354–3360. 10.1074/jbc.M307770200 14610090

[B27] Fraaije M. W. Kamerbeek N. M. van Berkel W. J. H. Janssen D. B. (2002). Identification of a Baeyer-Villiger monooxygenase sequence motif. *FEBS Lett.* 518 43–47. 10.1016/S0014-5793(02)02623-6 11997015

[B28] Fredricks K. M. (1967). Products of the oxidation of n-decane by *Pseudomonas aeruginosa* and *Mycobacterium rhodochrous*. *Antonie Van Leeuwenhoek* 33 41–48. 10.1007/BF02045532 4961926

[B29] Golyshin P. N. Martins Dos Santos V. A. P. Kaiser O. Ferrer M. Sabirova Y. S. Lünsdorf H. (2003). Genome sequence completed of *Alcanivorax borkumensis*, a hydrocarbon-degrading bacterium that plays a global role in oil removal from marine systems. *J. Biotechnol.* 106 215–220. 10.1016/j.jbiotec.2003.07.013 14651863

[B30] Golyshin P. N. Werner J. Chernikova T. N. Tran H. Ferrer M. Yakimov M. M. (2013). Genome sequence of *Thalassolituus oleivorans* MIL-1 (DSM 14913T). *Genome Announc.* 1:e0014113. 10.1128/genomeA.00141-13 23599290 PMC3630401

[B31] Grant C. Deszcz D. Wei Y.-C. Martínez-Torres R. J. Morris P. Folliard T. (2014). Identification and use of an alkane transporter plug-in for applications in biocatalysis and whole-cell biosensing of alkanes. *Sci. Rep.* 4:5844. 10.1038/srep05844 25068650 PMC5376172

[B32] Hand N. J. Klein R. Laskewitz A. Pohlschröder M. (2006). Archaeal and bacterial SecD and SecF homologs exhibit striking structural and functional conservation. *J. Bacteriol.* 188 1251–1259. 10.1128/JB.188.4.1251-1259.2006 16452406 PMC1367261

[B33] Hara A. Syutsubo K. Harayama S. (2003). Alcanivorax which prevails in oil-contaminated seawater exhibits broad substrate specificity for alkane degradation. *Environ. Microbiol.* 5 746–753. 10.1046/j.1468-2920.2003.00468.x 12919410

[B34] Harayama S. Kasai Y. Hara A. (2004). Microbial communities in oil-contaminated seawater. *Curr. Opin. Biotechnol.* 15 205–214. 10.1016/j.copbio.2004.04.002 15193328

[B35] Hardie K. R. Lory S. Pugsley A. P. (1996). Insertion of an outer membrane protein in *Escherichia coli* requires a chaperone-like protein. *EMBO J.* 15 978–988. 10.1002/j.1460-2075.1996.tb00434.x 8605893 PMC449992

[B36] Hazen T. C. Dubinsky E. A. DeSantis T. Z. Andersen G. L. Piceno Y. M. Singh N. (2010). Deep-sea oil plume enriches indigenous oil-degrading bacteria. *Science* 330 204–208. 10.1126/science.1195979 20736401

[B37] Hua F. Wang H. Q. (2014). Uptake and trans-membrane transport of petroleum hydrocarbons by microorganisms. *Biotechnol. Biotechnol. Equip.* 28 165–175. 10.1080/13102818.2014.906136 26740752 PMC4684044

[B38] Huysmans G. H. M. Guilvout I. Chami M. Nickerson N. N. Pugsley A. P. (2015). Lipids assist the membrane insertion of a BAM-independent outer membrane protein. *Sci. Rep.* 5:15068. 10.1038/srep15068 26463896 PMC4604470

[B39] Jain M. Cox J. S. (2005). Interaction between polyketide synthase and transporter suggests coupled synthesis and export of virulence lipid in *M. tuberculosis*. *PLoS Pathog.* 1:e2. 10.1371/journal.ppat.0010002 16201014 PMC1238737

[B40] Jeeves M. Knowles T. J. (2015). A novel pathway for outer membrane protein biogenesis in Gram-negative bacteria. *Mol. Microbiol.* 97 607–611. 10.1111/mmi.13082 26059329 PMC4973683

[B41] Ji Y. Mao G. Wang Y. Bartlam M. (2013). Structural insights into diversity and n-alkane biodegradation mechanisms of alkane hydroxylases. *Front. Microbiol.* 4:58. 10.3389/fmicb.2013.00058 23519435 PMC3604635

[B42] Kahng H. Y. Byrne A. M. Olsen R. H. Kukor J. J. (2000). Characterization and role of tbuX in utilization of toluene by *Ralstonia pickettii* PKO1. *J. Bacteriol.* 182 1232–1242. 10.1128/JB.182.5.1232-1242.2000 10671442 PMC94407

[B43] Kasai Y. Kishira H. Harayama S. (2002a). Bacteria belonging to the genus cycloclasticus play a primary role in the degradation of aromatic hydrocarbons released in a marine environment. *Appl. Environ. Microbiol.* 68 5625–5633. 10.1128/AEM.68.11.5625-5633.2002 12406758 PMC129893

[B44] Kasai Y. Kishira H. Sasaki T. Syutsubo K. Watanabe K. Harayama S. (2002b). Predominant growth of *Alcanivorax* strains in oil-contaminated and nutrient-supplemented sea water. *Environ. Microbiol.* 4 141–147. 10.1046/j.1462-2920.2002.00275.x 12000314

[B45] Kasai Y. Kishira H. Syutsubo K. Harayama S. (2001). Molecular detection of marine bacterial populations on beaches contaminated by the Nakhodka tanker oil-spill accident. *Environ. Microbiol.* 3 246–255. 10.1046/j.1462-2920.2001.00185.x 11359510

[B46] Klein B. Grossi V. Bouriat P. Goulas P. Grimaud R. (2008). Cytoplasmic wax ester accumulation during biofilm-driven substrate assimilation at the alkane–water interface by *Marinobacter hydrocarbonoclasticus* SP17. *Res. Microbiol.* 159 137–144. 10.1016/j.resmic.2007.11.013 18191384

[B47] Kostka J. E. Prakash O. Overholt W. A. Green S. J. Freyer G. Canion A. (2011). Hydrocarbon-degrading bacteria and the bacterial community response in gulf of Mexico beach sands impacted by the deepwater horizon oil spill. *Appl. Environ. Microbiol.* 77 7962–7974. 10.1128/AEM.05402-11 21948834 PMC3208977

[B48] Kotani T. Yurimoto H. Kato N. Sakai Y. (2007). Novel acetone metabolism in a propane-utilizing bacterium, *Gordonia* sp. strain TY-5. *J. Bacteriol.* 189 886–893. 10.1128/JB.01054-06 17071761 PMC1797311

[B49] Kryachko Y. Dong X. Sensen C. W. Voordouw G. (2012). Compositions of microbial communities associated with oil and water in a mesothermic oil field. *Antonie Van Leeuwenhoek* 101 493–506. 10.1007/s10482-011-9658-y 22038128

[B50] Lai Q. Li W. Shao Z. (2012). Complete genome sequence of *Alcanivorax dieselolei* type strain B5. *J. Bacteriol.* 194 6674–6674. 10.1128/JB.01813-12 23144414 PMC3497491

[B51] Letek M. González P. MacArthur I. Rodríguez H. Freeman T. C. Valero-Rello A. (2010). The genome of a pathogenic *Rhodococcus*: cooptive virulence underpinned by key gene acquisitions. *PLoS Genet.* 6:e1001145. 10.1371/journal.pgen.1001145 20941392 PMC2947987

[B52] Li H. Martin F. L. Zhang D. (2017). Quantification of chemotaxis-related alkane accumulation in acinetobacter baylyi using raman microspectroscopy. *Anal. Chem.* 89 3909–3918. 10.1021/acs.analchem.6b02297 28256129

[B53] Liu C. Wang W. Wu Y. Zhou Z. Lai Q. Shao Z. (2011). Multiple alkane hydroxylase systems in a marine alkane degrader, *Alcanivorax dieselolei* B-5. *Environ. Microbiol.* 13 1168–1178. 10.1111/j.1462-2920.2010.02416.x 21261799

[B54] Markovetz A. J. Kallio R. E. (1971). Subterminal oxidation of aliphatic hydrocarbons by microorganisms. *CRC Crit. Rev. Microbiol.* 1 225–237. 10.3109/10408417109104482 4950559

[B55] McKew B. A. Coulon F. Osborn A. M. Timmis K. N. McGenity T. J. (2007a). Determining the identity and roles of oil-metabolizing marine bacteria from the Thames estuary, UK. *Environ. Microbiol.* 9 165–176. 10.1111/j.1462-2920.2006.01125.x 17227421

[B56] McKew B. A. Coulon F. Yakimov M. M. Denaro R. Genovese M. Smith C. J. (2007b). Efficacy of intervention strategies for bioremediation of crude oil in marine systems and effects on indigenous hydrocarbonoclastic bacteria. *Environ. Microbiol.* 9 1562–1571. 10.1111/j.1462-2920.2007.01277.x 17504493

[B57] McKew B. A. Lefebvre S. C. Achterberg E. P. Metodieva G. Raines C. A. Metodiev M. V. (2013). Plasticity in the proteome of *Emiliania huxleyi* CCMP 1516 to extremes of light is highly targeted. *New Phytol.* 200 61–73. 10.1111/nph.12352 23750769

[B58] Minerdi D. Zgrablic I. Sadeghi S. J. Gilardi G. (2012). Identification of a novel Baeyer-Villiger monooxygenase from *Acinetobacter radioresistens*: close relationship to the *Mycobacterium tuberculosis* prodrug activator EtaA. *Microb. Biotechnol.* 5 700–716. 10.1111/j.1751-7915.2012.00356.x 22862894 PMC3815892

[B59] Mou X. Sun S. Edwards R. A. Hodson R. E. Moran M. A. (2008). Bacterial carbon processing by generalist species in the coastal ocean. *Nature* 451 708–711. 10.1038/nature06513 18223640

[B60] Narita S. Matsuyama S. Tokuda H. (2004). Lipoprotein trafficking in *Escherichia coli*. *Arch. Microbiol.* 182 1–6. 10.1007/s00203-004-0682-4 15221203

[B61] Nie Y. Fang H. Li Y. Chi C.-Q. Tang Y.-Q. Wu X.-L. (2013). The genome of the moderate halophile *Amycolicicoccus subflavus* DQS3-9A1(T) reveals four alkane hydroxylation systems and provides some clues on the genetic basis for its adaptation to a petroleum environment. *PLoS One* 8:e70986. 10.1371/journal.pone.0070986 23967144 PMC3743902

[B62] Nie Y. Liang J.-L. Fang H. Tang Y.-Q. Wu X.-L. (2014). Characterization of a CYP153 alkane hydroxylase gene in a Gram-positive *Dietzia* sp. DQ12-45-1b and its “team role” with alkW1 in alkane degradation. *Appl. Microbiol. Biotechnol.* 98 163–173. 10.1007/s00253-013-4821-1 23504079

[B63] Nikaido H. (2003). Molecular basis of bacterial outer membrane permeability revisited. *Microbiol. Mol. Biol. Rev.* 67 593–656. 10.1128/MMBR.67.4.593-656.2003 14665678 PMC309051

[B64] Nouwen N. Piwowarek M. Berrelkamp G. Driessen A. J. M. (2005). The large first periplasmic loop of SecD and SecF plays an important role in SecDF functioning. *J. Bacteriol.* 187 5857–5860. 10.1128/JB.187.16.5857-5860.2005 16077136 PMC1196081

[B65] Nouwen N. Ranson N. Saibil H. Wolpensinger B. Engel A. Ghazi A. (1999). Secretin PulD: association with pilot PulS, structure, and ion-conducting channel formation. *Proc. Natl. Acad. Sci. U.S.A.* 96 8173–8177. 10.1073/pnas.96.14.8173 10393967 PMC22207

[B66] Pacheco S. A. Hsu F.-F. Powers K. M. Purdy G. E. (2013). MmpL11 protein transports mycolic acid-containing lipids to the mycobacterial cell wall and contributes to biofilm formation in *Mycobacterium smegmatis*. *J. Biol. Chem.* 288 24213–24222. 10.1074/jbc.M113.473371 23836904 PMC3745366

[B67] Parales R. E. Harwood C. S. (2002). Bacterial chemotaxis to pollutants and plant-derived aromatic molecules. *Curr. Opin. Microbiol.* 5 266–273. 10.1016/S1369-5274(02)00320-X 12057680

[B68] Peterson J. A. Basu D. Coon M. J. (1966). Enzymatic omega-oxidation. I. Electon carriers in fatty acid and hydrocarbon hydroxylation. *J. Biol. Chem.* 241 5162–5164. 4380843

[B69] Pogliano J. A. Beckwith J. (1994). SecD and SecF facilitate protein export in *Escherichia coli*. *EMBO J.* 13 554–561. 10.1002/j.1460-2075.1994.tb06293.x 8313900 PMC394844

[B70] Postle K. Kadner R. J. (2003). Touch and go: tying TonB to transport. *Mol. Microbiol.* 49 869–882. 10.1046/j.1365-2958.2003.03629.x 12890014

[B71] Remans K. Pauwels K. van Ulsen P. Buts L. Cornelis P. Tommassen J. (2010). Hydrophobic surface patches on LolA of *Pseudomonas aeruginosa* are essential for lipoprotein binding. *J. Mol. Biol.* 401 921–930. 10.1016/j.jmb.2010.06.067 20620146

[B72] Reusch R. N. (2012). Insights into the structure and assembly of *Escherichia coli* outer membrane protein A. *FEBS J.* 279 894–909. 10.1111/j.1742-4658.2012.08484.x 22251410 PMC3392536

[B73] Riebel A. de Gonzalo G. Fraaije M. W. (2013). Expanding the biocatalytic toolbox of flavoprotein monooxygenases from *Rhodococcus jostii* RHA1. *J. Mol. Catal. B Enzym.* 88 20–25. 10.1016/j.molcatb.2012.11.009

[B74] Rojo F. (2009). Degradation of alkanes by bacteria. *Environ. Microbiol.* 11 2477–2490. 10.1111/j.1462-2920.2009.01948.x 19807712

[B75] Sabirova J. S. Becker A. Lünsdorf H. Nicaud J.-M. Timmis K. N. Golyshin P. N. (2011). Transcriptional profiling of the marine oil-degrading bacterium *Alcanivorax borkumensis* during growth on n-alkanes. *FEMS Microbiol. Lett.* 319 160–168. 10.1111/j.1574-6968.2011.02279.x 21470299

[B76] Schneiker S. dos Santos V. A. M. Bartels D. Bekel T. Brecht M. Buhrmester J. (2006). Genome sequence of the ubiquitous hydrocarbon-degrading marine bacterium *Alcanivorax borkumensis*. *Nat. Biotechnol.* 24 997–1004. 10.1038/nbt1232 16878126 PMC7416663

[B77] Seeliger J. C. Holsclaw C. M. Schelle M. W. Botyanszki Z. Gilmore S. A. Tully S. E. (2012). Elucidation and chemical modulation of sulfolipid-1 biosynthesis in *Mycobacterium tuberculosis*. *J. Biol. Chem.* 287 7990–8000. 10.1074/jbc.M111.315473 22194604 PMC3318749

[B78] Sillitoe I. Lewis T. E. Cuff A. Das S. Ashford P. Dawson N. L. (2015). CATH: comprehensive structural and functional annotations for genome sequences. *Nucleic Acids Res.* 43 D376–D381. 10.1093/nar/gku947 25348408 PMC4384018

[B79] Singer M. E. Finnerty W. R. (1984). *Microbial Metabolism of Straight-Chain and Branched Alkanes.* Available at: http://agris.fao.org/agris-search/search.do?recordID=US8605568

[B80] Singh S. N. Kumari B. Mishra S. (2012). *Microbial Degradation of Alkanes.* Berlin: Springer, 439–469. 10.1007/978-3-642-23789-8_17

[B81] Smits T. H. M. Witholt B. van Beilen J. B. (2003). Functional characterization of genes involved in alkane oxidation by *Pseudomonas aeruginosa*. *Antonie Van Leeuwenhoek* 84 193–200. 10.1023/A:1026000622765 14574114

[B82] Székely R. Cole S. T. (2016). Mechanistic insight into mycobacterial MmpL protein function. *Mol. Microbiol.* 99 831–834. 10.1111/MMI.13306 26710752

[B83] Tani A. Ishige T. Sakai Y. Kato N. (2001). Gene structures and regulation of the alkane hydroxylase complex in *Acinetobacter* sp. strain M-1. *J. Bacteriol.* 183 1819–1823. 10.1128/JB.183.5.1819-1823.2001 11160120 PMC95074

[B84] Teramoto M. Suzuki M. Okazaki F. Hatmanti A. Harayama S. (2009). Oceanobacter-related bacteria are important for the degradation of petroleum aliphatic hydrocarbons in the tropical marine environment. *Microbiology* 155 3362–3370. 10.1099/mic.0.030411-0 19541999

[B85] Throne-Holst M. Wentzel A. Ellingsen T. E. Kotlar H.-K. Zotchev S. B. (2007). Identification of novel genes involved in long-chain n-alkane degradation by *Acinetobacter* sp. strain DSM 17874. *Appl. Environ. Microbiol.* 73 3327–3332. 10.1128/AEM.00064-07 17400787 PMC1907095

[B86] Tokuda H. Matsuyama S. (2004). Sorting of lipoproteins to the outer membrane in *E. coli*. *Biochim. Biophys. Acta – Mol. Cell Res.* 1693 5–13. 10.1016/j.bbamcr.2004.02.005 15276320

[B87] Tsukahara J. Narita S.-I. Tokuda H. (2009). Real time analysis of lipoprotein transfer from LolA to LolB by means of surface plasmon resonance. *FEBS Lett.* 583 2987–2990. 10.1016/j.febslet.2009.08.032 19716823

[B88] van Beilen J. B. Funhoff E. G. (2007). Alkane hydroxylases involved in microbial alkane degradation. *Appl. Microbiol. Biotechnol.* 74 13–21. 10.1007/s00253-006-0748-0 17216462

[B89] van Beilen J. B. Funhoff E. G. van Loon A. Just A. Kaysser L. Bouza M. (2006). Cytochrome P450 alkane hydroxylases of the CYP153 family are common in alkane-degrading eubacteria lacking integral membrane alkane hydroxylases. *Appl. Environ. Microbiol.* 72 59–65. 10.1128/AEM.72.1.59-65.2006 16391025 PMC1352210

[B90] Van Beilen J. B. Li Z. Duetz W. A. Smits T. H. M. Witholt B. (2003). Diversity of alkane hydroxylase systems in the environment. *Oil Gas Sci. Technol.* 58 427–440. 10.2516/ogst:2003026

[B91] van Beilen J. B. Marín M. M. Smits T. H. M. Röthlisberger M. Franchini A. G. Witholt B. (2004). Characterization of two alkane hydroxylase genes from the marine hydrocarbonoclastic bacterium *Alcanivorax borkumensis*. *Environ. Microbiol.* 6 264–273. 10.1111/j.1462-2920.2004.00567.x 14871210

[B92] van Beilen J. B. Neuenschwander M. Smits T. H. M. Roth C. Balada S. B. Witholt B. (2002). Rubredoxins involved in alkane oxidation. *J. Bacteriol.* 184 1722–1732. 10.1128/JB.184.6.1722-1732.2002 11872724 PMC134906

[B93] van Beilen J. B. Panke S. Lucchini S. Franchini A. G. Röthlisberger M. Witholt B. (2001). Analysis of *Pseudomonas putida* alkane-degradation gene clusters and flanking insertion sequences: evolution and regulation of the alk genes. *Microbiology* 147 1621–1630. 10.1099/00221287-147-6-1621 11390693

[B94] van Beilen J. B. Penninga D. Witholt B. (1992). Topology of the membrane-bound alkane hydroxylase of *Pseudomonas oleovorans*. *J. Biol. Chem.* 267 9194–9201. 1315749

[B95] van Beilen J. B. Wubbolts M. G. Witholt B. (1994). Genetics of alkane oxidation by *Pseudomonas oleovorans*. *Biodegradation* 5 161–174. 10.1007/BF00696457 7532480

[B96] Van Bogaert I. N. A. Saerens K. De Muynck C. Develter D. Soetaert W. Vandamme E. J. (2007). Microbial production and application of sophorolipids. *Appl. Microbiol. Biotechnol.* 76 23–34. 10.1007/s00253-007-0988-7 17476500

[B97] van den Berg B. (2010). Going forward laterally: transmembrane passage of hydrophobic molecules through protein channel walls. *ChemBioChem* 11 1339–1343. 10.1002/cbic.201000105 20533493 PMC3013500

[B98] van den Berg B. Black P. N. Clemons W. M. Rapoport T. A. (2004). Crystal structure of the long-chain fatty acid transporter FadL. *Science* 304 1506–1509. 10.1126/science.1097524 15178802

[B99] Varela C. Rittmann D. Singh A. Krumbach K. Bhatt K. Eggeling L. (2012). MmpL genes are associated with mycolic acid metabolism in *Mycobacteria* and *Corynebacteria*. *Chem. Biol.* 19 498–506. 10.1016/j.chembiol.2012.03.006 22520756 PMC3370651

[B100] Vizcaíno J. A. Csordas A. del-Toro N. Dianes J. A. Griss J. Lavidas I. (2016). 2016 update of the PRIDE database and its related tools. *Nucleic Acids Res.* 44 D447–D456. 10.1093/nar/gkv1145 26527722 PMC4702828

[B101] Vila J. María Nieto J. Mertens J. Springael D. Grifoll M. (2010). Microbial community structure of a heavy fuel oil-degrading marine consortium: linking microbial dynamics with polycyclic aromatic hydrocarbon utilization. *FEMS Microbiol. Ecol.* 73 349–362. 10.1111/j.1574-6941.2010.00902.x 20528986

[B102] Wang L. Wang W. Lai Q. Shao Z. (2010). Gene diversity of CYP153A and AlkB alkane hydroxylases in oil-degrading bacteria isolated from the Atlantic Ocean. *Environ. Microbiol.* 12 1230–1242. 10.1111/j.1462-2920.2010.02165.x 20148932

[B103] Wang W. Shao Z. (2012). Diversity of flavin-binding monooxygenase genes (almA) in marine bacteria capable of degradation long-chain alkanes. *FEMS Microbiol. Ecol.* 80 523–533. 10.1111/j.1574-6941.2012.01322.x 22304419

[B104] Wang W. Shao Z. (2013). Enzymes and genes involved in aerobic alkane degradation. *Front. Microbiol.* 4:116 10.3389/fmicb.2013.00116 PMC3664771 23755043

[B105] Wang W. Shao Z. (2014). The long-chain alkane metabolism network of *Alcanivorax dieselolei*. *Nat. Commun.* 5:5755. 10.1038/ncomms6755 25502912

[B106] Wells R. M. Jones C. M. Xi Z. Speer A. Danilchanka O. Doornbos K. S. (2013). Discovery of a siderophore export system essential for virulence of *Mycobacterium tuberculosis*. *PLoS Pathog.* 9:e1003120. 10.1371/journal.ppat.1003120 23431276 PMC3561183

[B107] Wentzel A. Ellingsen T. E. Kotlar H.-K. Zotchev S. B. Throne-Holst M. (2007). Bacterial metabolism of long-chain n-alkanes. *Appl. Microbiol. Biotechnol.* 76 1209–1221. 10.1007/s00253-007-1119-1 17673997

[B108] Whyte L. G. Smits T. H. M. Labbé D. Witholt B. Greer C. W. van Beilen J. B. (2002). Gene cloning and characterization of multiple alkane hydroxylase systems in *Rhodococcus* strains Q15 and NRRL B-16531. *Appl. Environ. Microbiol.* 68 5933–5942. 10.1128/AEM.68.12.5933-5942.2002 12450813 PMC134402

[B109] Yakimov M. M. Denaro R. Genovese M. Cappello S. D’Auria G. Chernikova T. N. (2005). Natural microbial diversity in superficial sediments of Milazzo Harbor (Sicily) and community successions during microcosm enrichment with various hydrocarbons. *Environ. Microbiol.* 7 1426–1441. 10.1111/j.1462-5822.2005.00829.x 16104865

[B110] Yakimov M. M. Giuliano L. Crisafi E. Chernikova T. N. Timmis K. N. Golyshin P. N. (2002). Microbial community of a saline mud volcano at San Biagio-Belpasso, Mt. Etna (Italy). *Environ. Microbiol.* 4 249–256. 10.1046/j.1462-2920.2002.00293.x 12030850

[B111] Yakimov M. M. Giuliano L. Denaro R. Crisafi E. Chernikova T. N. Abraham W.-R. (2004). *Thalassolituus oleivorans* gen. nov., sp. nov., a novel marine bacterium that obligately utilizes hydrocarbons. *Int. J. Syst. Evol. Microbiol.* 54 141–148. 10.1099/ijs.0.02424-0 14742471

[B112] Yakimov M. M. Golyshin P. N. Lang S. Moore E. R. Abraham W. R. Lünsdorf H. (1998). *Alcanivorax borkumensis* gen. nov., sp. nov., a new, hydrocarbon-degrading and surfactant-producing marine bacterium. *Int. J. Syst. Bacteriol.* 48(Pt 2), 339–348. 10.1099/00207713-48-2-339 9731272

[B113] Yakimov M. M. Timmis K. N. Golyshin P. N. (2007). Obligate oil-degrading marine bacteria. *Curr. Opin. Biotechnol.* 18 257–266. 10.1016/j.copbio.2007.04.006 17493798

[B114] Yakimov M. M. Genovese M. Denaro R. (2010). “Thalassolituus,” in *Handbook of Hydrocarbon and Lipid Microbiology*, eds McGenity T. van der Meer J. R. de Lorenzo V. Timmis K. N. (Berlin: Springer-Verlag), 1764–1772.

[B115] Zhang Y. (2008). I-TASSER server for protein 3D structure prediction. *BMC Bioinformatics* 9:40 10.1186/1471-2105-9-0 PMC2245901 18215316

