# Supplementary material for: Differential Protein Expression During Growth on Medium Versus Long-Chain Alkanes in the Obligate Marine Hydrocarbon-Degrading Bacterium Thalassolituus oleivorans MIL-1
Source: Front Microbiol. 2018 Dec 17;9:3130. doi: 10.3389/fmicb.2018.03130 (PMC6304351; doi:10.3389/fmicb.2018.03130)
Supplement: Supplementary file 2 [file Data_Sheet_2.docx]

**Supplementary Figure 1** – Growth of Thalassolituus oleivorans MIL-1 at 16°C in ONR7a media enriched with aliphatic n-alkanes (*n*-C10-decane, *n*-C12-dodecane, *n*-C16-hexadecane, *n*-C20-eicosane, *n*-C24-tetracosane, *n*-C28-octacosane, n-C32-dotriacontane) or the branched alkane pristane at 0.1% (w/v) (means ± SE; n=3). Gas chromatography-mass spectrometry analysis (GC-MS) analysis revealed significant (P<0.05) alkane degradation in all single substrate microcosms after 21 days compared to the uninoculated control, ranging from 22% for *n*-C32 to 76% for *n*-C16.

**Supplementary Figure 2** - *Thalassolituus oleivorans* MIL-1 gene TOL_0709 codes for a Baeyer-Villiger monooxygenase (BVMO) class enzyme identified by two Rossmann fold motifs (GxGxx[G/A]) flanking two BVMO fingerprint sequence motifs. Conserved amino acids in the motif are shown with an asterisk *

- BVMO Motif 1: [A/G]GxWxxxx[F/Y]P[G/M]xxxD
- BMVO Motif 2: FxGxxxHxxxW[P/D], although the primary ‘F’ is occasionally substituted with a ‘Y’ mutation as observed here.

Image produced in Benchling (https://benchling.com/)

**Supplementary Figure 3** - Phylogenetic tree of class B flavoproteins. *Thalassolituus oleivorans* TOL_0709 (top of tree) groups within the BVMOs (Baeyer-Villiger Monooxygenases; Black). Also shown are Type 1 (Red) and Type II (Blue) FMOs (Flavin monooxygenases) and NMOs (*N*-hydroxylating monooxygenases; Green). Similar BVMOs identified from two other marine obligate hydrocarbonoclastic bacteria are also shown (*Oleispira* & *Oleibacter* species in bold). Other sequences obtained from the phylogenetic tree in Riebel *et al*. (2013). The evolutionary history was inferred using the Neighbour-Joining method [1]. The optimal tree with the sum of branch length = 1919 is shown. The tree is drawn to scale, with branch lengths in the same units as those of the evolutionary distances used to infer the phylogenetic tree. The evolutionary distances were computed using the number of differences method [2] and are in the units of the number of amino acid differences per sequence. Evolutionary analyses were conducted in MEGA6 [3].

1. Saitou N. and Nei M. (**1987**). The neighbor-joining method: A new method for reconstructing phylogenetic trees. *Molecular Biology and Evolution* **4**:406-425.

2. Nei M. and Kumar S. (**2000**). *Molecular Evolution and Phylogenetics*. Oxford University Press, New York.

3. Tamura K., Stecher G., Peterson D., Filipski A., and Kumar S. (**2013**). MEGA6: Molecular Evolutionary Genetics Analysis version 6.0. *Molecular Biology and Evolution*30: 2725-2729.

**Supplementary Figure 4** - Phylogenetic analysis of Etha and AlmA proteins. TOL_0709 long- chain alkane BMVO protein from this study is shown in bold and clusters with EthA type monoxygenases. The evolutionary history was inferred by using the Maximum Likelihood method based on the Le_Gascuel_2008 model [1]. The tree with the highest log likelihood (-15746.0620) is shown. The percentage of trees in which the associated taxa clustered together is shown next to the branches. Initial tree(s) for the heuristic search were obtained automatically by applying Neighbor-Join and BioNJ algorithms to a matrix of pairwise distances estimated using a JTT model, and then selecting the topology with superior log likelihood value. A discrete Gamma distribution was used to model evolutionary rate differences among sites (5 categories (+*G*, parameter = 0.8403)). The tree is drawn to scale, with branch lengths measured in the number of substitutions per site. There were a total of 342 positions in the final dataset. Evolutionary analyses were conducted in MEGA6 [2].

1. Le S.Q. and Gascuel O. (**1993**). An Improved General Amino Acid Replacement Matrix. *Mol Biol Evol* **25(7)**:1307-1320.

2. Tamura K., Stecher G., Peterson D., Filipski A., and Kumar S. (**2013**). MEGA6: Molecular Evolutionary Genetics Analysis version 6.0. *Molecular Biology and Evolution*30: 2725-2729.
